# Supplementary material for: Ultralight Open-Cell Graphene Aerogels with Multiple, Gradient Microstructures for Efficient Microwave Absorption
Source: Nanomaterials (Basel). 2022 Jun 1;12(11):1896. doi: 10.3390/nano12111896 (PMC9181992; doi:10.3390/nano12111896)
Supplement: Supplementary file 1 [file nanomaterials-12-01896-s001.zip › nanomaterials-1723808-supplementary.pdf]

nanomaterials

Supporting information

Ultralight Open-Cell Graphene Aerogels with Multiple, Gradient Microstructures for Efficient Microwave Absorption

Qilin Mei, Han Xiao, Guomin Ding *, Huizhi Liu, Chenglong Zhao, Rui Wang and Zhixiong Huang

School of Materials Science and Engineering, Wuhan University of Technology, 122 Luoshi Road, Wuhan 430070, China; meiqilin@whut.edu.cn (Q.M.); pxxiaohan@gmail.com (H.X.); ucchecknorth@gmail.com (H.L.) 246012@whut.edu.cn (C.Z.); 303842@whut.edu.cn (R.W.); zhixiongh@mail.whut.edu.cn (Z.H.)

* Correspondence: sdscdgm@126.com

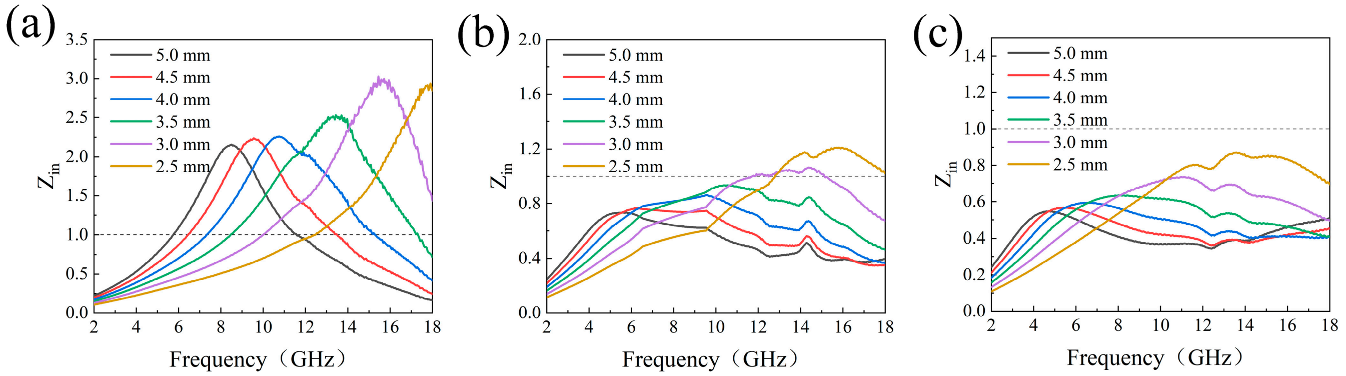

Figure S1. Normalized input impedance (Z_{in}) of (a) OCGA-L, (b) OCGA-M and (c) OCGA-H.

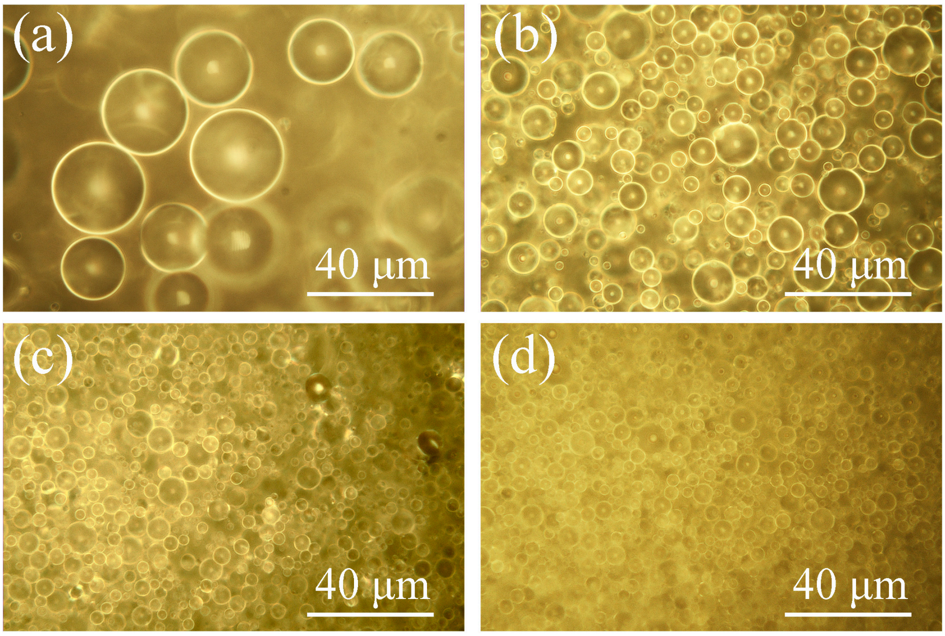

Figure S2. Ordinal micrographs of the emulsions prepared by applying rotational speeds of (a) 10k, (b) 15k, (c) 20k, and (d) 25k rpm, which had an average diameter of 30 μm, 10 μm, 6 μm and 4.5 μm, respectively. Apparently, the larger the rotational speed, the smaller the particle size.

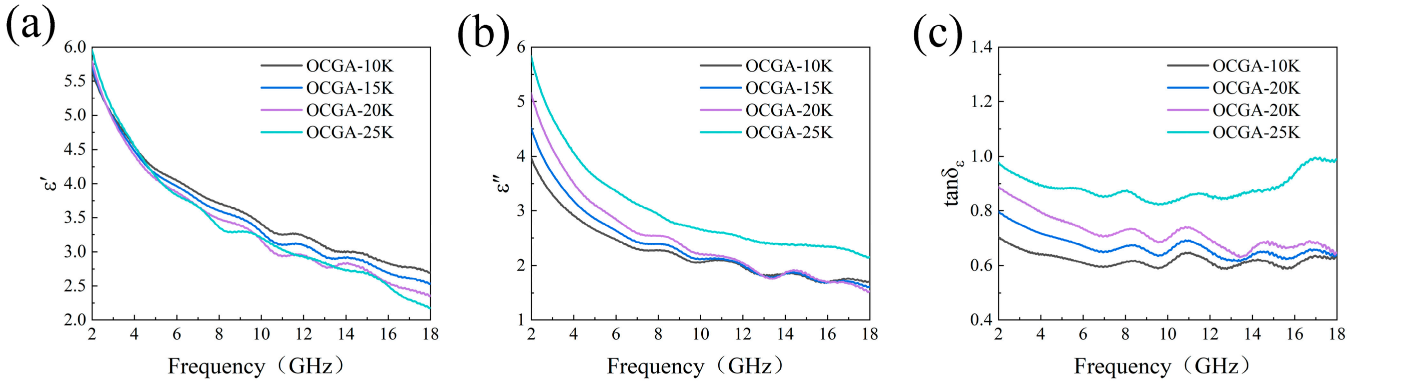

Figure S3. (a) Real part of permittivity(ε′), (b) imaginary part of permittivity(ε″) and (c) dielectric loss tangent (tanδ_ε) of OCGA-10K, OCGA-15K, OCGA-20K and OCGA-25K.

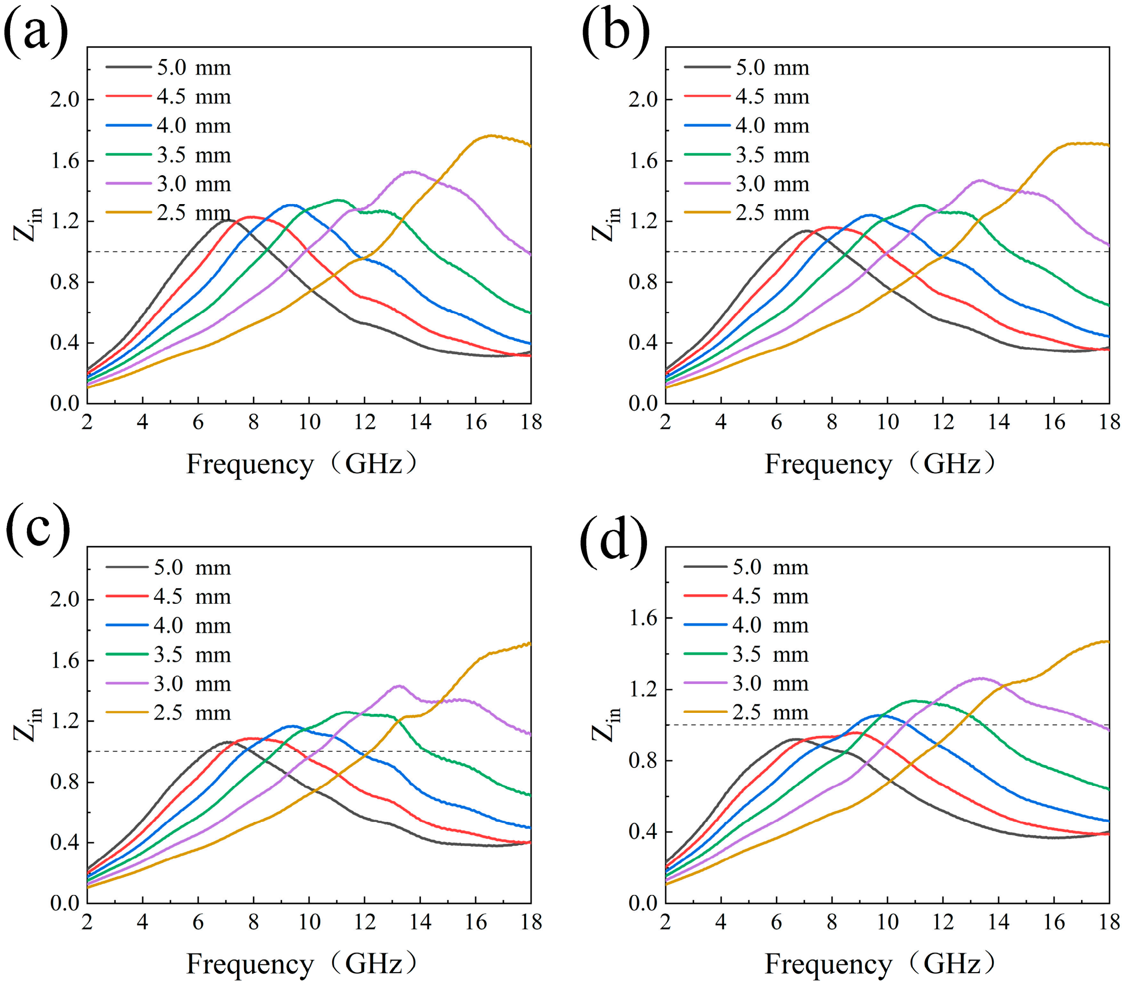

Figure S4. Normalized input impedance (Z_{in}) of (a) OCGA-10K, (b) OCGA-15K, (c) OCGA-20K and (d) OCGA-25K.

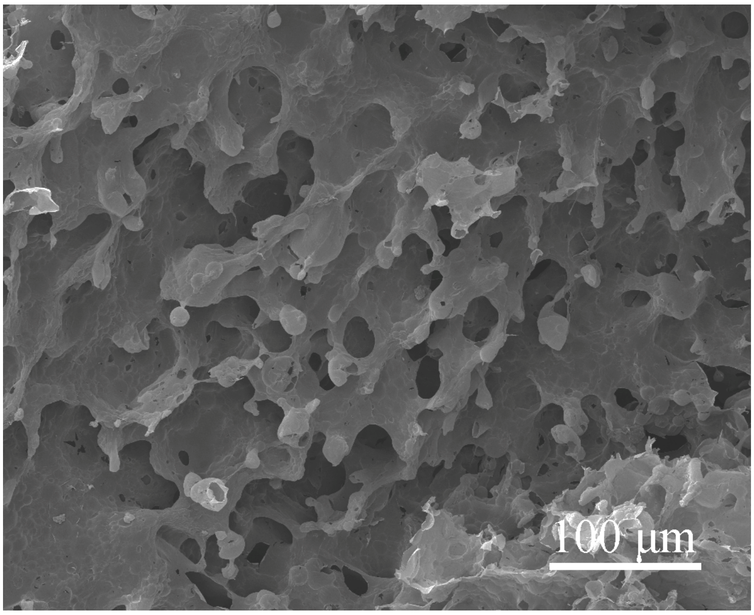

Figure S5. SEM images of OCGA-m.

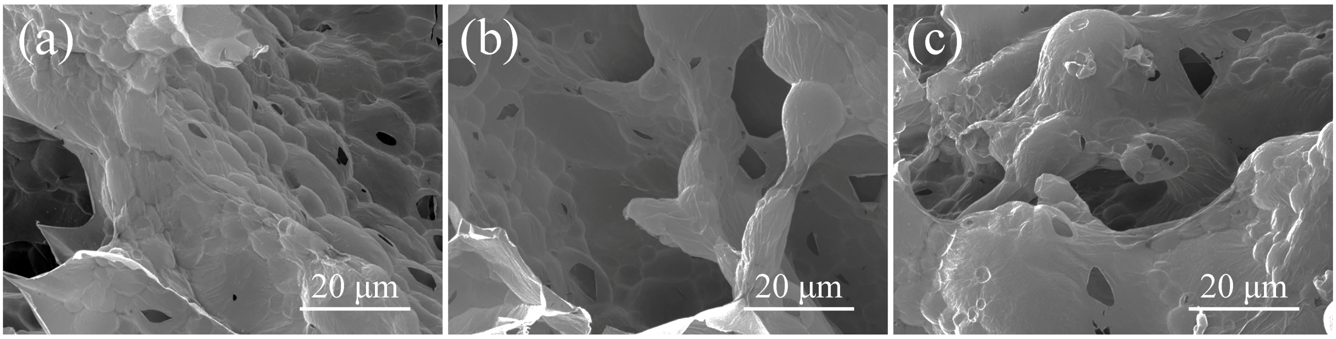

Figure S6. SEM images of (a) top region, (b) middle region and (c) bottom region of OCGA-P.

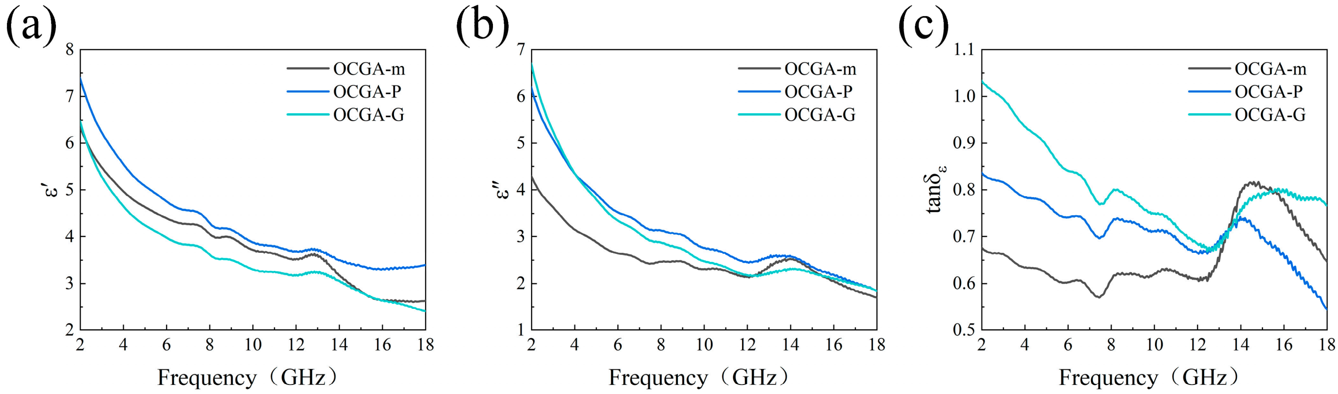

Figure S7. (a) Real part of permittivity(ε′), (b) imaginary part of permittivity(ε″) and (c) dielectric loss tangent ($tan\delta_{\varepsilon}$) of OCGA-m, OCGA-P, OCGA-G.

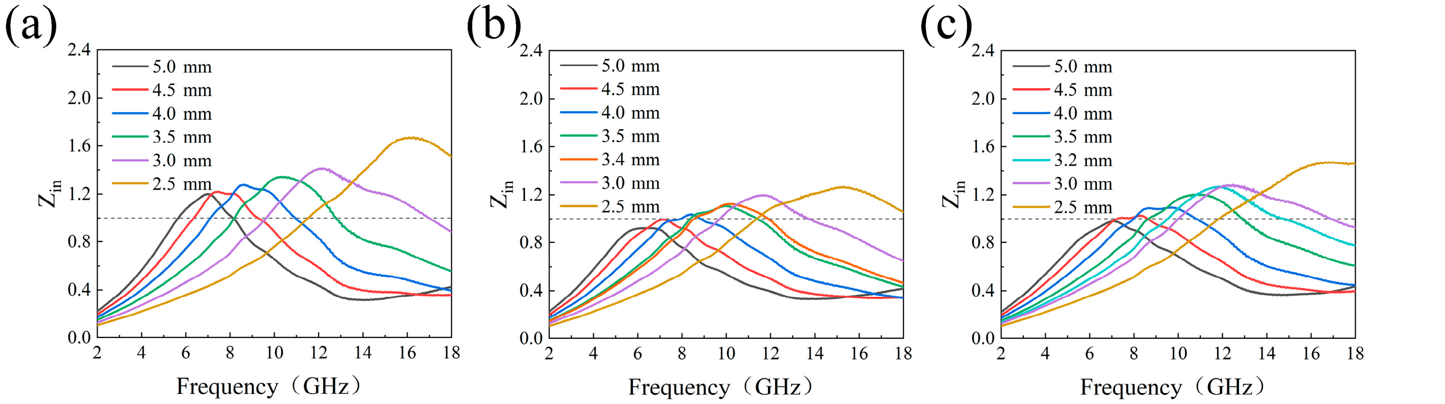

Figure S8. Normalized input impedance (Z_{in}) of (a) OCGA-10K, (b) OCGA-15K, (c) OCGA-20K and (d) OCGA-25K.
